# Skin Resident γδ T Cell Function and Regulation in Wound Repair

**DOI:** 10.3390/ijms21239286

**Published:** 2020-12-05

**Authors:** Luis D. Munoz, Michael J. Sweeney, Julie M. Jameson

**Affiliations:** Department of Biological Sciences, California State University San Marcos, San Marcos, CA 92096, USA; lmunoz@csusm.edu (L.D.M.); sween031@cougars.csusm.edu (M.J.S.)

**Keywords:** γδ T cell, T cell, wound repair, cytokine, chemokine, DETC, epidermis, dermis, diabetes, obesity

## Abstract

The skin is a critical barrier that protects against damage and infection. Within the epidermis and dermis reside γδ T cells that play a variety of key roles in wound healing and tissue homeostasis. Skin-resident γδ T cells require T cell receptor (TCR) ligation, costimulation, and cytokine reception to mediate keratinocyte activity and inflammatory responses at the wound site for proper wound repair. While both epidermal and dermal γδ T cells regulate inflammatory responses in wound healing, the timing and factors produced are distinct. In the absence of growth factors, cytokines, and chemokines produced by γδ T cells, wound repair is negatively impacted. This disruption in γδ T cell function is apparent in metabolic diseases such as obesity and type 2 diabetes. This review provides the current state of knowledge on skin γδ T cell activation, regulation, and function in skin homeostasis and repair in mice and humans. As we uncover more about the complex roles played by γδ T cells in wound healing, novel targets can be discovered for future clinical therapies.

## 1. Introduction

The skin acts as a barrier to prevent infection and limit mechanical damage. Protection at this epithelial barrier is mediated by the delicate crosstalk between keratinocytes and immune cells in order to maintain epidermal homeostasis and control infection. Skin-resident immune cell populations include αβ and γδ T cell receptor (TCR)-expressing T cells that reside in the epidermis and dermis of mammals with similarity to cells found in the skin of jawed vertebrates, suggesting evolutionary conservation [1,2,3]. While generally considered antimicrobial in function, skin-resident T cells also maintain homeostasis and support tissue repair [3,4,5,6,7,8,9,10,11,12]. In this review, we will focus on the role of T cells in wound repair and tissue maintenance with a special focus on γδ T cells.

In mice, epidermal γδ T cells express an invariant Vγ5Vδ1 TCR (Heilig and Tonegawa nomenclature [13]) which is specific for an unidentified antigen expressed by damaged or stressed keratinocytes [10,14,15,16]. Vγ5Vδ1 T cells develop in the fetal thymus on day 14 of fetal development [17]. After development, Vγ5Vδ1 T cells migrate to the epidermis, where they take on a dendritic morphology and are also known as dendritic epidermal T cells (DETC) [17]. Epidermal γδ T cells cannot be repopulated after birth, but instead undergo homeostatic proliferation within the basal layer of the epidermis to maintain their numbers [10,18]. Upon activation by damaged, stressed, or transformed keratinocytes, the epidermal γδ T cells lose their dendritic morphology and release growth factors, cytokines, and chemokines that assist in wound repair, protect from infection, and prevent malignancy [4,14,19,20,21].

Humans also have epidermal γδ T cells restricted to the Vδ1 TCR, but are polyclonal in nature and have an αβ T cell population present in the epidermis [5,22,23]. Human Vδ1 T cells respond rapidly to epidermal injury and produce insulin-like growth factor 1 (IGF-1) to facilitate wound closure and produce interferon-γ (IFN-γ) and tumor necrosis factor (TNF) in antitumor responses [5,8,24]. γδ T cells also populate the dermis of both mice and humans. In mice, over half of the dermal γδ T cells express the Vγ6Vδ1 TCR, while the rest express Vγ4Vδ4 [25,26]. Vγ4Vδ4 dermal T cells play key roles in wound repair by inducing a proinflammatory response via cytokine and chemokine secretion to recruit immune cells to the damaged site [25,26,27,28]. Humans have polyclonal Vδ1 γδ T cells in both the epidermis and dermis, making up approximately 10–20% of the T cells in human skin [5,29]. Upon activation, human dermal γδ T cells release cytokines including interleukin-17 (IL-17) and IL-22, to assist in inflammation [30]. Recently, skin T cells and their secreted products have become successful therapeutic targets for psoriasis [31,32], suggesting T cell regulation may also be considered for chronic nonhealing wounds.

Here, we will explore the roles played by epidermal and dermal γδ T cells in wound repair, with a particular focus on how common diseases such as obesity and diabetes impact T cell-mediated wound healing functions. It is important to understand the mechanisms by which T cells modulate wound repair in order to develop novel treatments to improve the healing of chronic wounds.

## 2. Epidermal T Cells, TCR Activation and Wound Repair

Epidermal γδ T cells recognize stressed or damaged keratinocytes via the γδ TCR [9,10,16]. While normally dendritic in morphology, activation by damaged neighboring cells causes epidermal γδ T cells to round up within 24 h after wounding [4]. Within 48 h, epidermal γδ T cells release keratinocyte growth factors 1 and 2 (KGF-1, KGF-2), which induce keratinocyte proliferation (Table 1) [4,33]. Epidermal γδ T cells regain their dendritic morphology 5 days post wounding [4]. Mice lacking γδ T cells exhibit a 2–3 day delay in wound closure and reduced epidermal hyperthickening, indicating that γδ T cells participate in wound repair [4]. γδ T cells also play key roles in burn wounds with TCRδ^−/−^ mice exhibiting a 50% increase in mortality postinjury [34]. In the absence of γδ T cells, both punch biopsy and burn wounds exhibit a delay in inflammation [20,35].

Epidermal γδ T cells have additional functions, such as protecting from infection, which can be especially important for chronic nonhealing wounds. Mice infected with *Staphylococcus aureus* (*S. aureus*) exhibit an expansion of γδ T cells in the skin-draining lymph nodes [48]. Expanded γδ T cells produce TNF/IFN-γ, which protect against subsequent infections, rather than IL-17A [48]. This expansion of TNF/IFN-γ-producing γδ T cells allows for healing of the skin lesions [48,49]. IFN-γ released by γδ T cells regulates neutrophils, recruiting them to the site of infection, assisting in the maturation and proliferation stages of wound repair [48]. In the lymph nodes, expanded γδ T cells express a CDR3 sequence of the TRGV5 gene that is identical to the invariant Vγ5^+^ TCR in epidermal γδ T cells of mice indicating clonotypic expansion to a similar antigen [48].

The γδ TCR is required for epidermal γδ T cell function and development, but it is less clear whether TCR-specificity is necessary for homing to the epidermis [10,17,18,50,51]. A keratinocyte antigen responsive γδ TCR is not required for epidermal γδ T cell homing, but interestingly enough, a keratinocyte responsive TCR is required for activation and long-term maintenance [10]. The epidermal γδ T cell TCR is persistently activated via the formation of phosphotyrosine-rich aggregates located on projections (PALPs) [52,53]. The PALPs act as anchors and polarize the cellular projections of the epidermal γδ T cells toward the apical surface of the epidermis [52,53]. After 48 h of stimulation, the TCR is internalized and the PALPs polarize the γδ T cell cellular projections [52,53]. This process acts as a long-range trans-epithelial transport to accumulate lysosomes at the apical barrier of the epidermis for possible TCR-recycling [52]. In addition to this, proper TCR signaling is required for γδ T cell development and maturation in the epidermis [50,51]. Lat deletion, specifically in adult γδ T cells, impairs TCR-dependent cytokine gene activation and the ability for epidermal γδ T cells to expand through proliferation [54]. Lat-deletion causes a delay in wound healing and impaired clonal expansion within the wound, thus TCR signaling via LAT is required for epidermal γδ T cell wound healing functions [54].

While the ligand for the epidermal γδ TCR remains elusive, butyrophilin and butyrophilin-like receptors regulate the development and localization of epithelial γδ T cells [55,56]. In mice, *Skint1* is expressed robustly in the skin and thymus and is critical in the thymic selection of Vγ5Vδ1 T cells [56]. In addition to this, *Skint2* is also expressed and is required for the maturation of epidermal γδ T cells [57]. The failure of Vγ5Vδ1 T cell development and maturation in the absence of Skints results in delayed wound repair [56,58,59]. Interestingly, mice deficient in other Skint genes also exhibit a delay in wound repair [59]. Specifically, knockdown of the *Skint3-4-9* gene cluster or epidermal deletion of *Skint3* or *9* results in delayed wound re-epithelialization [59]. While the butyrophilin-like *Skint1* gene resides in the human genome, it is not expressed due to premature termination codons in multiple frames; however, humans express butyrophilin genes that appear to play similar roles to Skints [56,58]. Therefore, it is suggested that butyrophilin and butyrophilin-like proteins play key roles in the regulation of epithelial γδ T cells in humans, as seen in the activation of intestinal γδ intraepithelial lymphocytes (IELs) [3,55,60]. In addition to butyrophilin and TCR requirements, epidermal γδ T cells require costimulation along with cytokine and chemokine signals to function as mediators of wound repair in the skin.

### 2.1. Impact of γδ T Cell Costimulation on Wound Repair

Epidermal γδ T cells require costimulation for full activation and function [43,44,61]. Several costimulatory receptors have been identified as modulators of epidermal γδ T cell activation including CD100, JAML, and NKG2D (Table 1). CD100 is expressed by epidermal γδ T cells and regulates activation by ligating plexin B2 on keratinocytes and inducing rounding and activation of epidermal γδ T cells [43]. CD100^−/−^ mice exhibit a two-day delay in wound repair, similar to TCRδ^−/−^ mice [4,43]. JAML activates epidermal γδ T cells through the ligand Coxsackie and Adenovirus receptor (CAR), inducing proliferation and production of IL-2, TNF, and KGF-1 [44]. When the JAML–CAR interaction is blocked immediately post wounding, epidermal γδ T cell activation is reduced at the wound edge and wound repair is delayed [44]. Costimulation through adhesion molecules is important for recruiting γδ T cells in wound repair. During wounding of the corneal epithelium, ICAM-1 is upregulated and is required for recruitment of γδ T cells to the site of damage in a lymphocyte function-associated antigen-1 (LFA-1)-dependent manner [62]. ICAM-1 deficiency in the epidermis leads to a delay in wound repair due to the inhibition of keratinocyte migration and formation of granulation tissue [61,63]. Thus, future studies should address whether ICAM-1 may be another receptor that costimulates epidermal or dermal γδ T cell wound repair functions in the skin [64].

NKG2D is an immunoreceptor highly expressed by epidermal γδ T cells that recognizes a series of receptors upregulated by stressed keratinocytes. NKG2D ligation is regulated via MHC class I-like molecules that induce functions such as cytolysis by adaptive immune cells [65]. H60 is a NKG2D ligand expressed in the skin by keratinocytes and functions to activate epidermal γδ T cells [45]. NKG2D ligands such as H60 are induced during tumorigenesis or infection, leading to activation of lymphocytes to lyse tumor cells and produce cytokines which protect the animal from malignancy or infection [66]. Aside from protection against malignancy and infection, H60 plays roles in wound repair. During wounding, H60 mRNA is upregulated, showing peak levels of mRNA on the first 2 days of wounding, indicating a role in wound repair [45]. When blocking H60 and NK2GD interactions, there is a delay in wound closure within the first 3 and 5 days, respectively [46,67]. In both mouse and human skin, the vast majority of γδ T cells express NKG2D, indicating H60–NK2GD interactions on epidermal γδ T cells are critical in wound repair [45]. Retinoic acid early inducible gene 1 (RAE-1) is another NKG2D ligand expressed in malignancy and is induced in healing wounds [21,67]. RAE-1 ligation of NKG2D costimulates epidermal γδ T cell degranulation, IL-2 production, and proliferation [47]. It is still controversial whether NKG2D engagement alone is enough to trigger lysis or whether TCR engagement is required [45,47,68]. Taken together, costimulation through cellular receptors carefully regulates the complex functions of epidermal γδ T cells in the skin; however, additional regulation occurs via secreted products such as cytokines and chemokines.

### 2.2. Cytokine and Chemokine Regulation of Skin γδ T Cells

Cytokine regulation is critical for efficient epidermal γδ T cell localization, homeostatic turnover, and downstream function in the skin. In the absence of common γ-chain cytokines, such as IL-2, IL-7, IL-4, and IL-15, epidermal γδ T cells are diminished in number (Table 2) [69,70,71,72]. IL-2 and IL-7 are required for epidermal γδ T cell proliferation [72] and IL-7R is required for the induction of rearrangement and transcription of the TCR-γ chain [73]. IL-4 and IL-15 both promote growth of epidermal γδ T cells, but IL-15 also regulates epidermal γδ T cell survival [72,74]. Furthermore, signaling through IL-15Rα regulates the development of IL-17-producing γδ T cells (Tγδ17 cells) [42]. In addition to IL-15Rα, IL-1β and IL-23 can also stimulate γδ T cells to produce IL-17 [40,75]. Production of IL-17A is critical in playing selective roles in wound repair [76]. In IL-17A^−/−^ mice, there is a delay in wound repair that can be restored in skin organ cultures by the addition of wild type epidermal γδ T cells [19]. Beyond the complex array of cytokines required for skin γδ T cell development, growth, survival, and function, chemokines regulate migration and recruitment.

**Figure 1:**
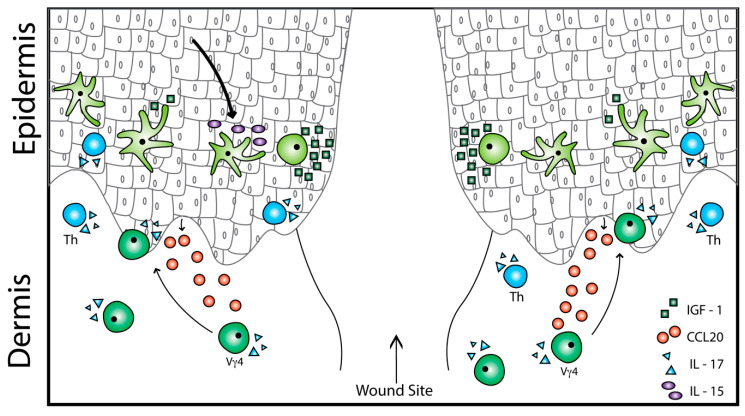
Typical cellular crosstalk occurring in murine wound repair. During wound repair, interleukin-15 (IL-15) is secreted by keratinocytes to activate epidermal γδ T cells to release insulin-like growth factor-1 (IGF-1) and prevent keratinocyte apoptosis. Chemokine ligand 20 (CCL20) is released by keratinocytes to recruit dermal γδ T cells expressing IL-17A and induce local inflammation. IL-17A is also released by epidermal γδ T cells to activate proliferation, differentiation, and migration of keratinocytes.

In early development, fetal thymic epidermal γδ T cell precursors and recently recruited epidermal γδ T cells in mice express GPR15, an orphan G protein-linked chemoattractant receptor, that regulates γδ T cell recruitment to the skin during development [85]. In the absence of GPR15, there is a delay in the recruitment of epidermal γδ T cells to the skin [85]. Five days after birth, Chemokine receptor 4 (CCR4) and CCR10 are able to partially recover epidermal γδ T cells over time [81,85]. CCR10 is required for homing of epidermal γδ T cells to the skin, and upon arrival, CCR4 is required for γδ T cells to replenish themselves by self-renewal [81,85]. On the other hand, in the dermis, dermal γδ T cells express the chemokine CCR6 and, upon stress in the epidermis, are recruited into the epidermis by chemokine ligand 20 (CCL20) (Figure 1) [27,40,88]. During inflammation, CCL20 production by keratinocytes is upregulated by IL-17A, IL-22, IL-23, and TNF-α, increasing epidermal infiltration by dermal γδ T cells [27,87,89]. In the absence of CCR6, wounds have a 4 day delay in wound closure and fewer γδ T cells at the wound site, indicating a key role for CCR6 in efficient wound repair [84]. The CCL20–CCR6 axis of dermal T cell recruitment occurs similarly in the human epidermis, resulting in Th17 cell infiltration [87,90]. Together, homeostatic and inflammatory chemokine production is precisely regulated for γδ T cell homing and localization for mediating efficient wound repair.

### 2.3. Epidermal γδ T Cell Function in Wound Repair

After wounding, epidermal γδ T cells release a variety of growth factors, cytokines, and chemokines to assist in wound repair [4,91,92,93]. The release of KGFs by epithelial γδ T cells is critical for repair in both the skin and intestine [4,94]. KGF-1 binds to keratinocytes via FGFR2-IIIb and induces keratinocyte proliferation and hyaluronan production [36,91]. During wounding, KGF-1 produced by epidermal γδ T cells induces keratinocytes to deposit hyaluronan in the extracellular matrix [35]. Upon KGF-1 binding, keratinocytes upregulate hyaluronan synthesis (HAS) through the expression of hyaluronan synthesis-2 (HAS2) and hyaluronan synthesis-3 (HAS3) [91]. Hyaluronan participates in the inflammation associated with wound repair through facilitating leukocyte infiltration [91]. As hyaluronan is produced by keratinocytes, macrophages are recruited and infiltrate the wound site [35].

γδ T cells produce additional factors in response to tissue damage in both mice and humans, including IGF-1 and IL-17 [5,7,90,95]. IGF-1 provides a survival signal preventing keratinocytes and epidermal γδ T cells from undergoing apoptosis [7]. Murine models have shown that production of IGF-1 by epidermal γδ T cells is regulated through the secretion of IL-15 by keratinocytes [37]. If IL-15 is blocked, then IGF-1 secretion is reduced, resulting in delayed wound healing [37]. To further assist in wound repair, epidermal γδ T cells release IL-17A that induces the production of epidermal antimicrobial peptides such as β-defensins 3, regenerating islet-derived protein 3γ, and S100A8 [19,96,97,98]. This production of antimicrobial peptides induces proliferation and differentiation of keratinocytes and regulates keratinocyte migration [19,96,97,98,99]. Similar results have been reported in murine burn wounds, where increased activation of IL-17^+^ and IL-10^+^ γδ T cells induces keratinocyte proliferation and monocyte recruitment to the wound site, respectively [80,100]. While epidermal γδ T cells recruit macrophages, neutrophils, and myeloid cells to assist with wound repair, γδ T cells from the dermis are also recruited to the epidermis to participate in wound healing.

## 3. Dermal γδ T Cell Activation in Inflammation and Repair

In mice, there are two main populations of γδ T cells in the dermis—Vγ6 T cells that reside in the dermis and Vγ4 T cells that recirculate. Vγ6 T cells are a highly homogenous Scart1^+^ population unlike Vγ4 T cells that are Scart2^+^ [101]. Vγ6 T cells act as persistent effector cells in the skin. This persistence is due to Vγ6 T cells highly expressing the anti-apoptotic Bcl2a1 protein that allows protection against activation-induced cell death [101]. This allows Vγ6 T cells to proliferate, terminally differentiate, and migrate to the site of action while leaving behind a small population of anti-apoptotic Vγ6 T cells [101]. On the other hand, Vγ4 T cells represent 20% of dermal γδ T cells and are migratory. Vγ4 T cells utilize CCR6 for recruitment to the epidermis. CCR6 expressed on Vγ4 T cells binds to CCL20, which is expressed by epidermal keratinocytes, endothelial cells, and dendritic cells during skin inflammation [27,28,102]. This allows the highly motile Vγ4 T cells to infiltrate the epidermis and assist during wound repair [27,28,102]. Vγ4 T cells are most commonly found early post wounding, making up half of the IL-17A^+^ cells on day 3 [103]. Neutralizing CCL20 reduces the number of Vγ4 T cells infiltrating the epidermis at the wound site, thereby reducing IL-17A production and overall inflammation [103].

In major injuries of the skin caused by trauma or necrosis, damage-associated molecular patterns (DAMPs) are generated by mitochondria and are capable of activating dermal γδ T cells [104]. Interestingly enough, these DAMPs are not generated upon controlled circumstances such as cell death and apoptosis, indicating a specific use of DAMPs [105]. DAMPs are critical in activating the innate immune system via pattern recognition receptors (PPRs), activating toll-like receptors (TLRs) to assist in inflammation, infection, and skin injury [106,107]. In the presence of activated mitochondrial DAMPs, γδ T cells increase cytokine release in a correlative manner [108]. When dermal γδ T cells are activated, they release IL-17A and induce local inflammation. Long after skin inflammation has subsided, Vγ4 T cells are still able to persist in the skin by clonally expanding after inflammation subsides [109]. After inflammation, these clonally expanded Vγ4 T cells are able to quickly respond to the recurring stress and expand throughout the skin to newly affected sites previously not challenged [109]. Upon IL-17A release by dermal γδ T cells, there is enhanced IL-23/IL-1β expression that inhibits IGF-1 production in epidermal γδ T cells [103]. This direct inhibition of IGF-1 by IL-23/IL-1β leads to delayed wound healing in the epidermis due to reduced keratinocyte proliferation [103]. This negative correlation between IL-23/IL-1β expression and IGF-1 production allows for regulation of local inflammation and keratinocytes proliferation to maintain skin homeostasis. On the other hand, if there is a disruption in skin homeostasis due to disease, the mechanisms used to repair wounds will be affected.

## 4. Obesity, Type 2 Diabetes, and Skin T Cells

In both mice and humans, obesity and type 2 diabetes result in impaired wound healing [93,110,111,112,113,114,115,116,117]. The underlying molecular and cellular interactions in diabetic nonhealing wounds are important to uncover in order to advance clinical treatments and therapies. Skin γδ T cells are among the cells rendered dysfunctional as mice and humans become obese and develop diabetes [93,114,115,118]. The number, homeostatic turnover, and function of γδ T cells are all negatively impacted, causing defects in barrier function, skin homeostasis, and wound closure [5,93,114,118,119,120,121]. As hyperglycemia becomes overt in db/db mice, the number of epidermal γδ T cells becomes reduced by half due to reduced homeostatic turnover [93]. Similar to TCRδ^−/−^ mice, obese and diabetic mice exhibit a thinner epidermis with a reduced capacity for maintaining skin homeostasis due to premature differentiation and reduced proliferation of keratinocytes [121]. In addition, obesity and type 2 diabetes render epidermal γδ T cells dysfunctional with regard to growth factor and cytokine production at the wound site [93].

**Figure 2:**
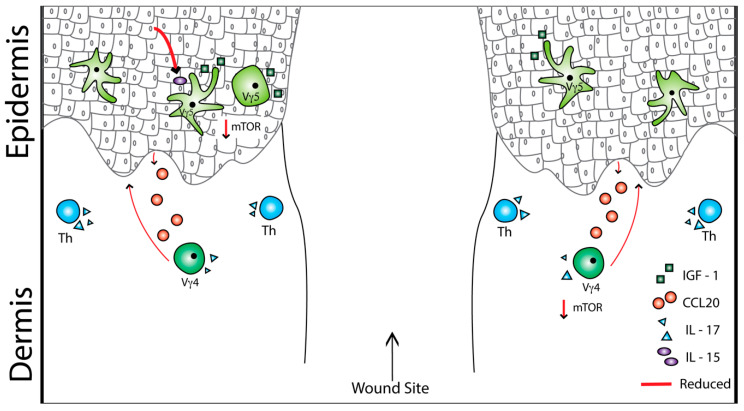
Obesity and type 2 diabetes cause alterations in cellular crosstalk that result in delayed wound repair. Epidermal γδ T cells become reduced in number, causing premature keratinocyte differentiation, epidermal thinning, and reduced production of interleukin-15 (IL-15) and IL-17. Upon wounding, the decreased number and function of γδ T cells result in a reduction in insulin-like growth factor-1 (IGF-1) and decreased keratinocyte proliferation. In addition, less chemokine ligand 20 (CCL20) is produced by keratinocytes, hindering the recruitment of dermal γδ T cells to the epidermis. Together, this leads to a delay in wound repair.

The mechanisms of epidermal γδ T cell dysfunction in obesity and type 2 diabetes are complex and still being uncovered. Chronic inflammation plays an important role as blocking TNF with antibodies prior to wound generation in diabetic, obese mice improves epidermal γδ T cell growth factor and cytokine production at the wound site [93,122]. Furthermore, chronic inflammation modulates tissue residence of γδ T cell populations in other epithelial tissues such as the intestine [123,124]. In addition to chronic inflammation, a role for Aryl hydrocarbon receptor (AHR) signaling is likely involved. AHR signaling has important roles in the health and disease of the immune system and is generally involved in cellular metabolism. AHR signaling, which is required for epidermal γδ T cells to properly develop, becomes reduced during obesity [116,125]. In the absence of AHR, there are 50% fewer epidermal γδ T cells and the remaining γδ T cells express higher levels of inflammatory genes such as *IFN-γ*, *granzyme F (GZMF)*, and *programmed death-ligand 1 (PDL-1)* [125]. AHR signaling reduces inflammation and maintains homeostasis in the skin by inhibiting inflammatory genes and upregulating cell morphology and ion homeostasis genes [125]. Upregulation of cell morphology F-actin enzymes Advillin and Fermt2 and ion homeostasis ion channels Kcnab and Kcnma1 allows for proper epidermal γδ T cell activation and downstream proliferation [125]. Obesity reduces AHR signaling, causing fewer epidermal γδ T cells to round up and release cytokines upon activation, which leads to a delay in wound repair [116,125]. Another mechanism of epidermal γδ T cell dysfunction in obesity and diabetes is the disruption of the IL-15-IGF-1 loop (Figure 2). In diabetic mice and humans, there is reduced IGF-1 production at the wound site [112,113]. During wound repair, epidermal γδ T cells interact with keratinocytes to initiate an IL-15-IGF loop, which amplifies IGF-1 production for re-epithelization [37,126]. In addition, diabetes can impair the mTOR pathway, causing a reduction in IL-15 activation, leading to a reduction in IGF-1 and wound closure [126]. Reduced IGF-1 production negatively impacts keratinocyte proliferation, leading to a delay in re-epithelization for wound closure [112,113].

Type 1 diabetes also impacts γδ T cells and wound repair functions. In streptozotocin (STZ)-induced diabetic mice, IL-7 is reduced, leading to impaired dermal Vγ4 T cell maintenance [126]. Furthermore, CCL20/CCR6 chemokine signaling is weakened, leading to a reduction in the recruitment of dermal Vγ4 T cells post wounding (Figure 2). STZ-induced diabetic mice also exhibit reduced levels of IL-23, a major activator for Vγ4 T cells, and IL-1β in the dermis adjacent to the wound, resulting in diminished IL-17 production [115]. Normally, IL-1β and IL-23 inhibit IGF-1 production by epidermal γδ T cells; however, in diabetic mice, the addition of IL-17A and IL-15 improves wound healing by forming positive loops that enhance local inflammation and re-epithelization, respectively [103,114,115]. Interestingly, in type 2 diabetic mice, inhibiting IL-17A and IL-23 improves the rate of wound closure over 14 days [127]. This indicates that a delicate balance of IL-17A and IL-23 is required to avoid chronic wounds. This balance is correlated with the delay in wound closure, suggesting that Vγ4 T cells may play a key role in chronic wounds.

There are fewer skin-resident γδ T cells in diabetic humans with chronic wounds than in non-diabetic humans, suggesting that the findings in mice are relevant to humans [12]. Obesity also reduces the number and impairs the antiviral function of γδ T cells in the peripheral blood of humans [117]. In chronic nonhealing wounds, skin-resident γδ T cells are impaired, leading to decreased levels of growth factors such as IGF-1 in chronic wounds of diabetic and obese humans [5,112]. Epidermal Vδ1^+^ T cells normally produce IGF-1 to promote wound healing and in the dermis, can induce angiogenesis through a vascular endothelial growth factor (VEGF)-dependent pathway [5,128]. However, in chronic wounds of patients, IGF-1 production by γδ T cells is reduced in the skin [5,112]. In addition, epidermal Vδ1^+^ T cells produce less IL-2 and cannot be rescued by stimulation with ionomycin, indicating that the γδ T cells in chronic wounds are less responsive to activation [5]. With a decrease in IL-2 production and activation, epidermal γδ T cells in chronic wounds are less likely to activate, proliferate, and orchestrate wound repair, which may contribute to problems with wound healing [5].

## 5. Conclusions

The epidermis and dermis comprise γδ T cells that are critical in orchestrating key aspects of wound healing. Epidermal γδ T cells provide essential cytokines and growth factors that regulate epidermal homeostasis, but also promote inflammation at the wound site. Dermal γδ T cells provide cytokines and chemokines that regulate inflammation throughout the skin, and feedback on epidermal γδ T cells. Given this, epidermal and dermal γδ T cells promote a complex crosstalk with keratinocytes and inflammatory cells to provide balance and maintain skin homeostasis. As wounding occurs, dermal γδ T cells are recruited by keratinocytes to orchestrate inflammation. Simultaneously, epidermal γδ T cells release growth factors and cytokines to orchestrate keratinocyte re-epithelialization and inflammation.

Obesity and diabetes disrupt the precise timing and impact of the delicate crosstalk between γδ T cells and keratinocytes. As obesity progresses, the skin exhibits defects in barrier function, homeostasis, and wound closure. The impact on γδ T cells is profound with defects in homeostatic turnover and cytokine/growth factor production. As scientists elucidate the molecular and cellular interactions at play with γδ T cells in diabetic nonhealing wounds, it opens doors for the advancement of clinical treatments and therapies for chronic wounds.

## Figures and Tables

**Table 1:** Factors, cytokines, and receptors that regulate and are regulated by γδ T cells in the skin.

Factors/Cytokines/Receptors	Function in Wound Repair	Reference
Keratinocyte Growth Factor-1 (KGF-1)	Induces keratinocyte proliferation, migration, and differentiation.	[33,36]
Insulin-like Growth Factor-1 (IGF-1)	Facilitates wound closure by mediating keratinocyte survival and limiting differentiation along with enhancing migration.	[36,37,38]
Tumor Necrosis Factor (TNF)	Induces inflammation.	[39]
Interleukin-17A (IL-17A)	Induces inflammation and the proliferation and differentiation of keratinocytes.	[40,41,42]
CD100	Regulates activation through ligation of plexin B2.	[43]
Junction Adhesion Molecule-like Protein (JAML)	Costimulates proliferation and cytokine/growth factor production.	[44]
Natural Killer Group 2D (NKG2D)	Recognizes receptors upregulated by stressed keratinocytes and induces cytolysis.	[45]
H60	Upregulated upon stress and ligates NKG2D to activate γδ T cells.	[46]
Retinoic Acid Early Inducible 1 (Rae-1)	Ligates NKG2D to costimulate epidermal γδ T cell degranulation, Il-2 production, and proliferation.	[47]

**Table 2:** Cytokines and chemokines that regulate and are regulated by γδ T cells in the skin.

Cytokine/Chemokine/Receptor	Function in Wound Repair	Reference
Interleukin-2 (IL-2)	Induces proliferation of epidermal γδ T cells	[77]
IL-7	Induces proliferation of epidermal γδ T cells and TCR-γ chain rearrangement	[78]
IL-4	Influences number and growth of γδ T cells	[79]
IL-15	Regulates growth and survival of γδ T cells and production of IGF-1	[37]
IL-23	Recruits dermal γδ T cells, stimulates IL-17 production, and inhibits IGF-1 production	[31]
IL-10	Reduces inflammatory responses	[80]
Chemokine Receptor 4 (CCR4)	Recruits γδ T cells to wound site or site of infection	[81,82]
CCR6	Binds CCL20 to recruit γδ T cells from the dermis to the epidermis	[83,84]
CCR10	Binds CCL27 for homing of γδ T cells to the epidermis	[85,86]
Chemokine Ligand 20 (CCL20)	Binds CCR6 to induce γδ T cell recruitment from the dermis to epidermis.	[87]
